# Evolution of methane permeability and fracture structure of coal: implications for methane extraction in deep coal seams

**DOI:** 10.1039/d2ra05811a

**Published:** 2022-10-24

**Authors:** Wenqiang Ju, Jun Dong, Chenxu Chang

**Affiliations:** College of Safety Science and Engineering, Nanjing Tech University Nanjing Jiangsu 211816 China dong_jun@njtech.edu.cn; Jiangsu Key Laboratory of Urban and Industrial Safety, Nanjing Tech University Nanjing Jiangsu 211816 China; State Key Laboratory Cultivation Base for Gas Geology and Gas Control (Henan Polytechnic University) Jiaozuo Henan 454000 China

## Abstract

A traditional view is that gas in the intact coal is easier to be extracted compared with tectonic coal after stress relief. With the increase in buried depth, however, the structure of coal and the permeability evolution law may change. Thus, the variation of permeability characteristics and the efficiency of gas extraction method at a deeper coal seam need further study. In this paper, the intact coal and tectonic coal in the Qinan coal mine are taken as the research objects. The permeabilities of both coal samples were tested under hydrostatic pressure, and the effective stress was loaded to a high level and then unloaded to the initial value. The differences of fracture structure between the intact coal and tectonic coal were also analyzed. The results show that, after loading and unloading, the permeability recovery rate of the intact coal is lower than that of the tectonic coal, and the fracture loss degree is higher than that of the tectonic coal. The reason is that the contact area between the matrixes of intact coal is relatively small, and the high stress leads to the plastic deformation or fracture of the matrix rock bridge. The contact area between the matrixes of tectonic coal is much larger and has a stronger resistance to compression deformation. The fracture structure of intact coal makes it more difficult to extract gas under high stress conditions. It is necessary to combine various stress relief methods such as protective layer mining and hydraulic punching to expand the fracture aperture, and enhance the permeability and the gas extraction efficiency.

## Abstract 2

Permeability of tectonic coal is easier to recover after loading and unloading, indicating that its fracture structure is more stable than that of the intact coal under high stress conditions.

## Graphical abstract

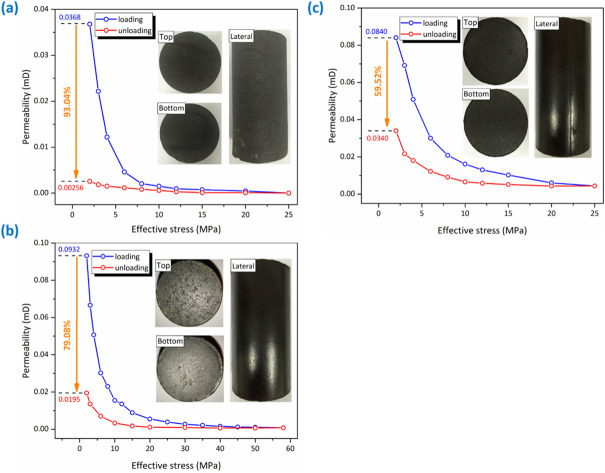

## 1. Introduction

Coal mine gas, composed mainly of methane, is a main factor that seriously endangers the safe production of coal. Gas accidents mainly include gas explosions, coal and gas outbursts, gas combustion and gas asphyxiation.^1–3^ With the increasing emphasis on gas disasters and the advances in gas control technology, some achievements have been made in gas disaster prevention, but coal mine gas accidents continue to happen. Methane is an efficient and clean energy with a calorific value of 35.9 MJ m^−3^, equal to the calorific value of 1.2 kg of standard coal.^4^ Methane is also a greenhouse gas, and its emission is not eco-friendly. The extraction and utilization of gas in coal seams can not only ensure the safe exploitation of mineral resources and promote the utilization of coal mine gas, but also protect the atmospheric environment, which can help to achieve triple benefits of safety, energy and environment.^5–7^

With the increase in the depth of coal mining, the geostress rises, and the permeability of coal seam decreases gradually. Thus, the difficulty of gas extraction keeps rising, and the risk of gas disaster intensifies continuously. Many scholars have investigated the gas permeability characteristics of coal under different stress states and gas pressure conditions. Meng *et al.* found that the permeability of high-order coals altered dramatically when the effective confining stress varied in the range of 2.5–10 MPa. When the effective confining pressure is higher than 10 MPa, the permeability curve tends to be flat.^8^ Tian carried out loading and unloading experiments under an effective stress of 0–10 MPa. After stress unloading, the permeability of tectonic coal only recovered to 25% of the initial value, which was much lower than that of non-tectonic coal.^9^ Wang *et al.* concluded that the permeability of coal is closely related to its mechanical characteristics and is greatly affected by geostress.^10^ Mckee *et al.* calculated the relationship between the coal seam permeability and the burial depth in several basins in the United States, which revealed that the permeability decreased exponentially with the increase in the burial depth and the effective stress.^11^ Cheng *et al.* concluded that the *in situ* permeability of tectonic coal was obviously lower than that of intact coal, while the opposite result was obtained in the laboratory. The main reason is that the tectonic coal samples reconstructed in the laboratory may not be appropriate, and the research on gas extraction and utilization of tectonic coal remains to be further developed.^12^

The research of scholars has enriched the gas migration characteristics and permeability evolution law, which played a positive role in guiding efficient gas extraction. It is commonly recognized that the permeability of intact coal is large, and the coal is always in the elastic deformation stage. The fractures of coal will be opened after stress relief, and the gas is easy to be extracted. On the contrary, the tectonic coal undergoes plastic deformation, and the gas is difficult to be extracted.^12–14^

However, previous studies mostly focused on the stress smaller than 10 MPa, which means that shallow coal seams were tested. With the increase in burial depth, the fracture structure and the permeability evolution law may vary because of the higher geostress. Thus, whether the intact coal is still in the elastic deformation state under the high stress condition, and whether the gas can be extracted efficiently after unloading needs further discussion. Furthermore, most scholars focus on the permeability evolution law of the intact coal. The research on the permeability characteristics of the tectonic coal and its relationship with that of the intact coal is insufficient. Therefore, the permeability characteristics of the intact and tectonic coal under high stress loading–unloading process need to be further studied.

In this study, the hydrostatic pressure was loaded to a high value and then unload, and the permeability characteristics of the intact coal and tectonic coal were tested. The relationship of the permeability characteristics between the intact coal and the tectonic coal were investigated. Furthermore, the differences of fracture structure between the intact coal and tectonic coal were further analyzed. Finally, the technical suggestions for enhancing the effect of methane extraction in deep coal seams were proposed. The research work in this paper can enrich the gas permeability characteristics of deep coal seams, which is of great significance to gas disaster prevention, resource utilization, and environmental protection.

## 2. Coal specimen preparation and permeability test

### 2.1. Coal specimen preparation

**Fig. 1:**
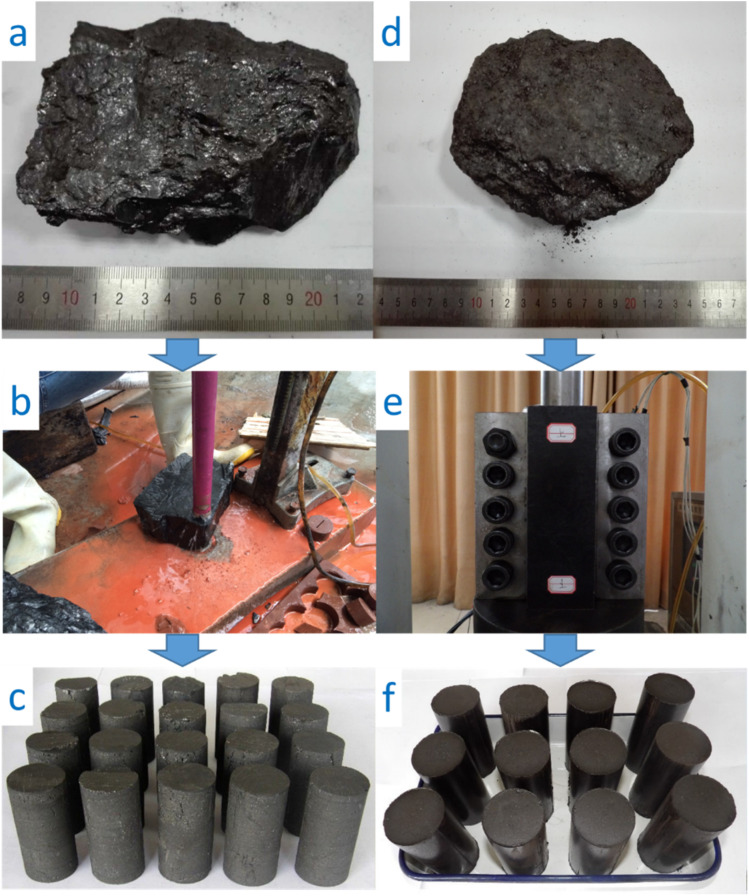
Preparation of intact and tectonic coal samples and formed specimens.

The samples of this study were collected from the Qinan coal mine in the Chinese Huaibei coalfield. The photos of the intact coal and tectonic coal were taken, as shown in Fig. 1a and d, respectively. The intact coal block was drilled (Fig. 1b), and then cut and polished to form the specimens (*ϕ* 50 × 100 mm, Fig. 1c). A mold and a servo press were used to compress the tectonic coal into the cylindrical samples under the stress of 150 MPa for ten hours (Fig. 1e), then the specimens can be obtained after demoulding, as shown in Fig. 1f.

### 2.2. Methane permeability tests

**Fig. 2:**
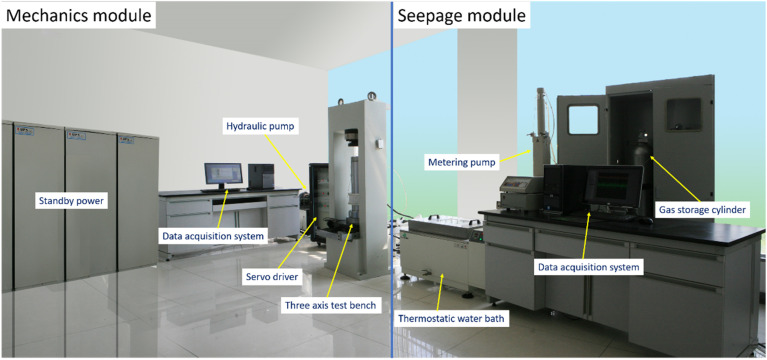
Illustration of the absorption-seepage-mechanical coupling testing system.

The methane permeability of coal is a key parameter affecting the migration and extraction of gas in coal seams.^15–17^ The absorption-seepage-mechanical coupling testing system was adopted to obtain the methane permeability of the standard coal specimens, and the physical diagram of the device is illustrated in Fig. 2.

The permeability testing methods mainly consist of the steady-state method and the transient method. When the permeability of the tested specimen is smaller than 0.1 mD, the transient method should be used.^12^ The permeability of coal samples used in the experiment is low, so the transient pressure pulse method is used for the permeability test in this paper.

On the basis of Darcy's law as eqn (1).1
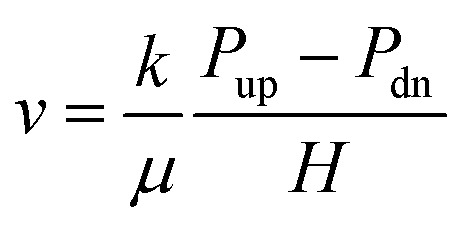


Ignoring the compression storage of fluid in the coal samples, the relationship for this condition can be obtained as eqn (2).2
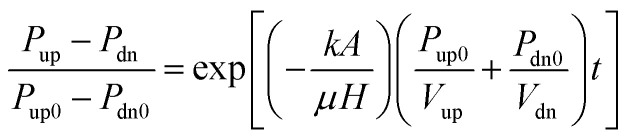
where *v* – fluid flow rate in coal sample, m s^−1^; *k* – permeability of coal samples, m^2^, 1 mD = 0.987 × 10^−15^ m^2^; *μ* – fluid dynamic viscosity, Pa s; *P*_up_ – upstream fluid pressure of coal sample, Pa; *P*_dn_ – downstream fluid pressure of coal sample, Pa; *P*_up0_ – upstream fluid pressure of coal sample at initial time, MPa; *P*_dn_ – downstream fluid pressure of coal sample, MPa; *P*_dn0_ – downstream fluid pressure of coal sample at initial time, MPa; *V*_up_ – total volume of upstream gas storage tanks and pipelines, m^3^; *V*_dn_ – total volume of downstream gas storage tanks and pipelines, m^3^; *H* – height of coal sample, m; *A* – cross-sectional area of coal sample, m^2^; *t* – testing time, s.

**Fig. 3:**
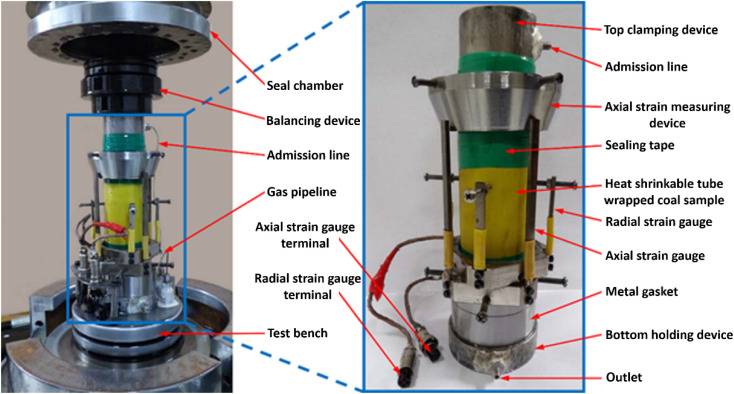
Coal sample installation of permeability test.

The main steps of the permeability test are:

(1) Place the sample gently on the holding device, and place the top clamping device on the upper end of the coal sample. Use a heat gun to make close contact between the heat shrink tube and the coal sample.

(2) Install the coal sample on the test bench, turn on the test system. The installed coal sample is shown in Fig. 3.

(3) Seal the triaxial pressure chamber, inject hydraulic oil, turn on the high-precision temperature control system after oil injection and adjust to 30 °C, and then apply 3 MPa hydrostatic pressure to the coal sample.

(4) Vacuum the coal sample for 48 h, and inject methane pressure of 1 MPa into the system using the metering pump until the equilibrium state.

(5) Then the value of permeability can be calculated according to eqn (2).

## 3. Results and discussions

### 3.1. Permeability characteristics during hydrostatic loading and unloading

**Fig. 4:**
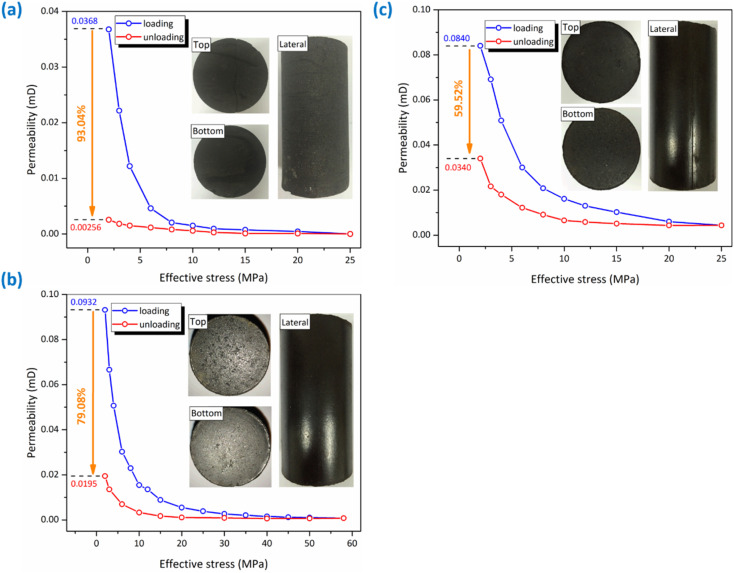
Permeability testing results of the intact coal (a), tectonic coal A (b) and tectonic coal B (c) during hydrostatic loading and unloading process.

A relatively complete standard sample of the intact coal was selected for the permeability test. The relationship between the permeability and the effective stress under the hydrostatic loading and unloading conditions is illustrated in Fig. 4a. The permeability of intact coal sample decreases continuously during the loading process, mainly due to the narrowing of seepage channel caused by the compression of the fracture. When the effective stress is 2 MPa, the permeability value is 0.0368 mD. During the loading process of effective stress from 2 MPa to 8 MPa, the permeability decreases rapidly and then slowly with the increase in the effective stress. When the effective stress reaches 25 MPa, the transient method is difficult to obtain the effective methane permeability, and the calculated permeability value is only 1.41 × 10^−5^ mD. Since the permeability value cannot be effectively measured under the effective stress of 25 MPa, it is of no practical significance to further load this intact coal sample, so the permeability test under hydrostatic pressure and pressure relief is carried out subsequently. With the decreasing effective stress, the permeability increases slowly. When the effective stress is unloaded to 2 MPa, the permeability value is only 2.56 × 10^−3^ mD. The permeability of the intact coal sample can only recover to 6.96% of the initial value after loading and unloading. The permeability does not show a significant trend of increase, indicating that permanent damage occurs under the hydrostatic loading process.

A standard sample of the tectonic coal was firstly selected for the permeability test to obtain the relationship between the permeability and the effective stress during hydrostatic loading and unloading process, as shown in Fig. 4b. Similar to the permeability evolution law of the intact coal, the permeability of the tectonic coal decreases continuously during the loading process. When the effective stress is 2 MPa, the permeability is 0.0932 mD. During the loading process of effective stress from 2 MPa to 25 MPa, the permeability decreases rapidly and then declines slowly with the increasing effective stress. When the effective stress is loaded to 25 MPa, the obvious permeability can still be measured, which is 3.86 × 10^−3^ mD. Then, higher hydrostatic pressure was applied to the coal sample, and more permeability values were tested during loading process. With the further increase in the effective stress, it is found that the permeability decreases slowly. Because the maximum confining pressure of the experimental equipment is 60 MPa, hydrostatic pressure of 59 MPa was chosen as the maximum value applied to the tectonic coal specimen (effective stress of 58 MPa), and the permeability value at this time is 7.62 × 10^−4^ mD.

Since the loading stress could not continue to increase, the permeability test during hydrostatic unloading was then conducted. From Fig. 4b, the permeability increases slowly with the decreasing effective stress at first, and then a rapid increase occurs when the effective stress is smaller than 10 MPa. When the effective stress is reduced to 2 MPa, the permeability value is 0.0195 mD. The permeability of the tectonic coal sample recovers to 20.92% of the initial value after loading and unloading.

The loading and unloading stress paths of the tectonic coal samples are different from those of the intact coal. It is necessary to compare the permeability characteristics of the two coal samples, so another tectonic coal sample is selected to carry out the permeability test under the same mechanical path as that of the intact coal. The tectonic coal sample that has been tested is named tectonic coal A, and the newly selected tectonic coal sample is named tectonic coal B. The tectonic coal collected is pulverized, and tectonic coal A and B are remodeled under the same condition, so they have no essential difference. The relationship between the permeability and the effective stress during hydrostatic loading and unloading is shown in Fig. 4c. Similar to the permeability evolution law of the intact coal and tectonic coal A, the permeability of tectonic coal B decreases continuously during the loading process. When the effective stress is 2 MPa, the permeability is 0.0840 mD, which is slightly lower than that of the tectonic coal A. When the effective stress reaches 25 MPa, the permeability is 4.37 × 10^−3^ mD, slightly larger than that of the tectonic coal A. The permeability evolution law of the tectonic coal A is consistent with the tectonic coal B, and the difference in values between them is unobvious, indicating that the discreteness of the tectonic coal samples formed by remodeling is small. After the effective stress reaches 25 MPa, the permeability is tested under hydrostatic unloading process. From Fig. 4c, with the decrease in the effective stress, the permeability increases slowly at first and then rapidly when the effective stress is less than 10 MPa. When the effective stress is unloaded to 2 MPa, the permeability is 0.0340 mD. After loading and unloading, the permeability of the tectonic coal B recovers to 40.48% of the initial value, and the recovery rate of permeability is much larger than that of the tectonic coal A.

### 3.2. Comparison of the relative permeability

**Fig. 5:**
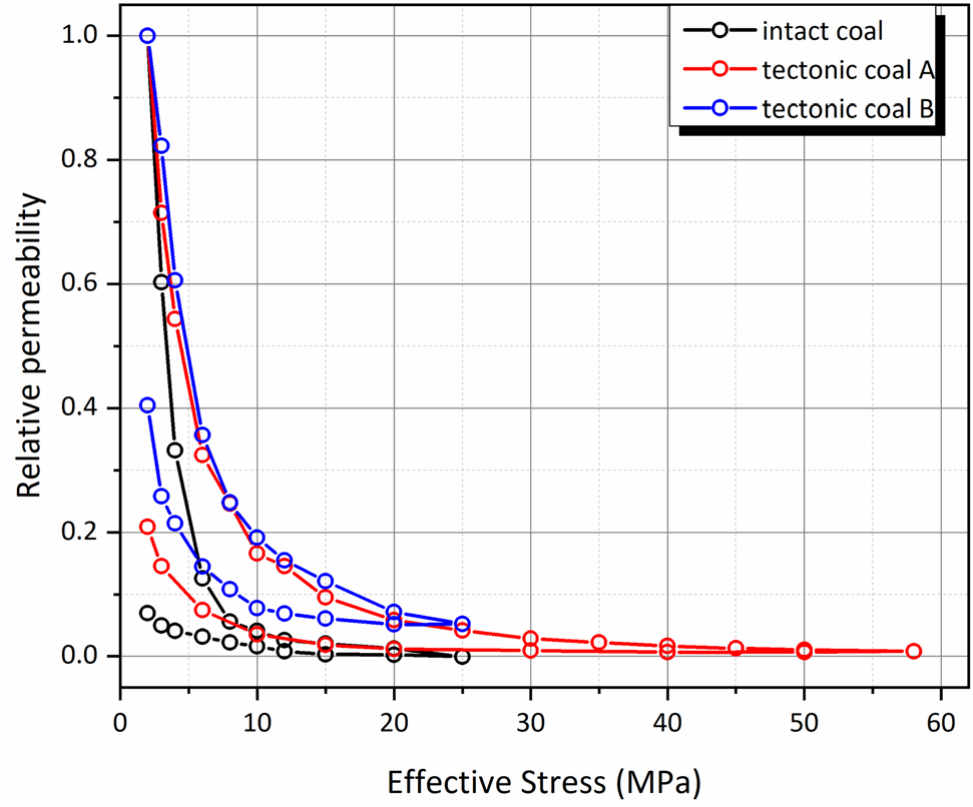
Relationship between the relative permeability and the effective stress of the three coal samples.

The permeability of methane in coal is significantly affected by the scale of fracture, and the permeability of the intact coal sample is discrete. Thus, it may be better to compare the ratio of the permeability value under a certain stress condition to that under the initial effective stress of 2 MPa (defined as relative permeability in this paper). The relationship between the relative permeability and the effective stress of the three coal samples are illustrated in Fig. 5.

The relative permeability reduction laws of the two tectonic coals are almost the same, but the value of the tectonic coal B is higher than that of the tectonic coal A during the unloading process. Considering that the permeability evolution law of the loading process is similar when the effective stress is smaller than 25 MPa, it indicates that loading the tectonic coal A to a high stress condition (59 MPa) causes more damage to the fracture structure, resulting in a smaller recovery rate.

By comparing the relative permeability of the intact coal and the tectonic coal B, it is found that the reduction rate of the relative permeability for the intact coal is much larger than that for the tectonic coal B, and the recovery capacity during the unloading process is much lower. Thus, it can be concluded that the fracture of intact coal is prone to damage under the condition of high stress, while the fracture structure of the tectonic coal is more stable.

### 3.3. Comparison of the loss degree of fracture

To better characterize the variation of the fracture scale for different coal samples after loading and unloading, the loss degree of fracture is calculated in the following part. The intact coal has obvious parallel plate shaped fractures, which are the main seepage channel. The opening size determines the permeability of fracture. Poiseuille derived the permeability of a smooth parallel plate, which can be expressed as eqn (3):^18^^,^^19^3
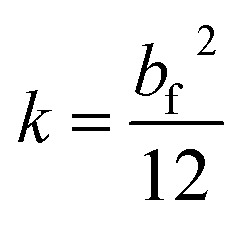
where, *b*_f_ – aperture of fracture, m.

Under *in situ* conditions, the tectonic coal particles are closely contacted with each other, accompanied by elastic–plastic deformation. The unbonded portions between the particles can be treated as capillary fractures. Assuming that the capillary is a cylinder, according to Hagen–Poiseuille's law, the permeability through circular fractures is shown as eqn (4):^20^4
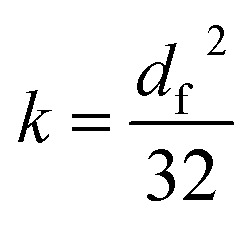
where *d*_f_ – diameter of fracture, m.

Usually, the cross-section of the fracture may be closer to a square under the effect of stress.^21^ When the fracture section is square, setting the hydraulic radius equals to that of a circular pipe, then the permeability of gas flowing through square fractures is shown as eqn (5):5
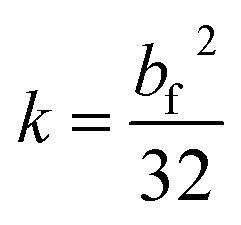
where *b*_f_ – edge length of the square section fracture, m.

**Fig. 6:**
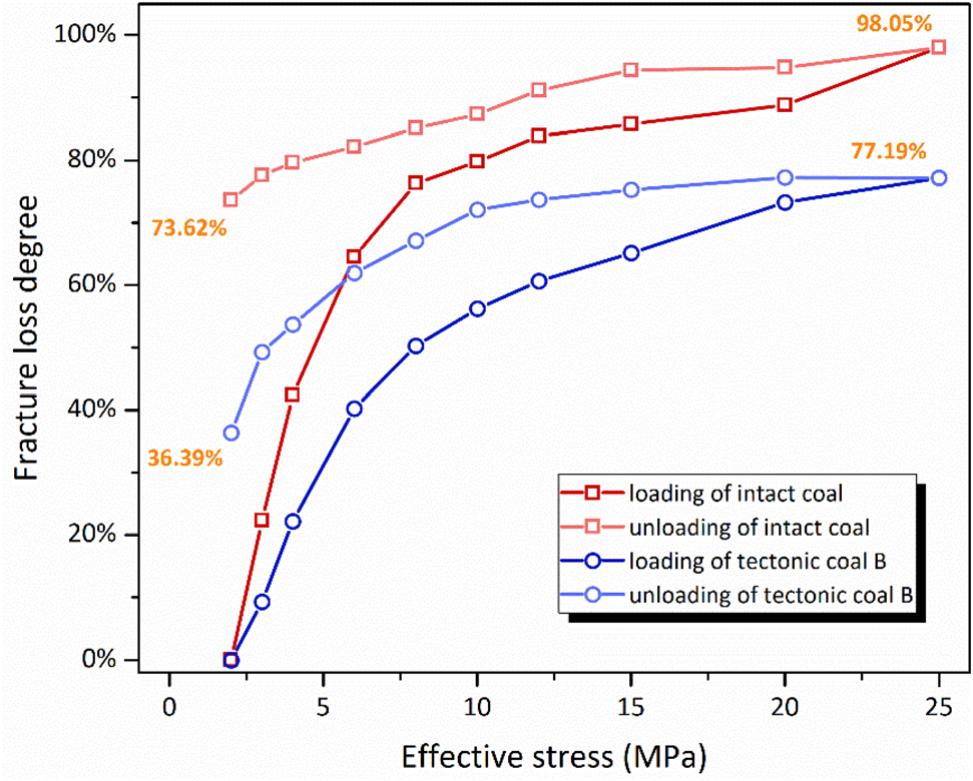
Comparative analysis of fracture loss degree between intact coal and tectonic coal B.

From the above equations, it can be founded that the permeability is proportional to the square of fracture size. Then the fracture loss degrees of the intact coal and the tectonic coal B can be calculated, as shown in Fig. 6. The loading and unloading path of intact coal is 2 MPa → 25 MPa → 2 MPa. When the effective stress is loaded to 25 MPa, the permeability is merely 1.41 × 10^−5^ mD, and the fracture loss degree is up to 98.05%. After loading and unloading, the fracture loss degree is 73.62%, meaning that the majority of the fractures are permanently damaged. As for the tectonic coal with the same mechanical path as the intact coal, the fracture loss degree is 36.39% after loading and loading. Thus, it can be concluded that the fracture structure for the tectonic coal has a stronger ability to resist deformation under the higher stress condition.

Studies have shown that the matrix strength of intact coal is greater than that of the tectonic coal.^22^ We found that the recovery of relative permeability of tectonic coal sample is higher than that of intact coal, and the fracture loss degree is lower than that of the intact coal. This indicates that the seepage channels of intact coal and tectonic coal are different, *i.e.*, their fracture structures are inconsistent.

### 3.4. Evolution of coal fracture structure

**Fig. 7:**
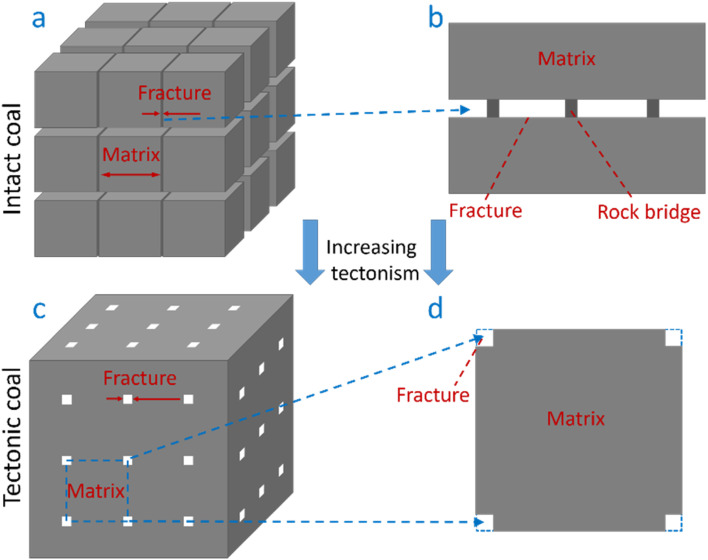
Evolution of the matrix and fracture with increasing tectonism.

The differences in the permeability evolution law and fracture loss degree between the intact and tectonic coal depend on their fracture structure.^23–25^ The physical structure model of intact coal is usually considered as a cube model, and the fractures are parallel plate shaped (Fig. 7a).^5^ The adjacent matrix blocks are connected by the rock bridges with some area in the fractures (Fig. 7b). Due to the small opening of fracture and the large strength of matrix rock bridge, elastic deformation occurs under lower stress, and the permeability can recover to the initial value after unloading. However, the matrix rock bridge could not resist the compression of higher stress because the contact area between the matrix blocks is small.^26^ High effective stress may cause the plastic deformation or even breakage to the rock bridges, resulting in the loss of the fracture aperture and a significant reduction in the permeability.

With the enhancement of tectonism, the intact coal is gradually broken and pulverized to form tectonic coal. The powders are gradually bonded under the effects of geostress and temperature, causing the formation of columnar fracture channel (Fig. 7c).^27^ The structure of the tectonic coal can be idealized as matrixes connecting with each other, and the fractures are located at the edges of the cubic matrix (Fig. 7d).

Although the strength of the tectonic coal particles is weaker than that of intact coal, the contact area of the matrix is larger, so it has a stronger resistance to the compression of stress. Thus, the tectonic coal has a smaller fracture loss degree and higher permeability recovery rate under the effect of high stress.

### 3.5. Implications for methane extraction in deep coal seams

**Fig. 8:**
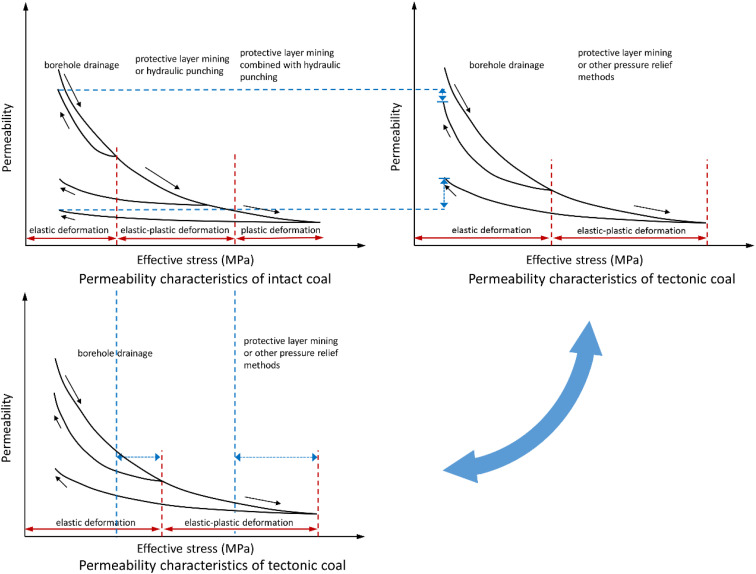
Comparison of the permeability evolution and gas extraction method between the intact and tectonic coal under different effective stress.

The permeability evolution laws and the fracture structures of the intact and tectonic coal are analyzed by laboratory measurements under the loading and unloading paths. The results show that coal seams under different depths are in the different stages of deformation. To achieve better extraction, different stress relief measures should be selected pertinently. Combined with the above analysis and previous studies, the concept figure of permeability characteristics of the intact coal and tectonic coal varying with effective stress can be obtained, as illustrated in Fig. 8.

The results show that high stress leads to the plastic deformation for the structure of intact coal, and the permeability is difficult to recover to the loading value after unloading. Meanwhile, the tectonic coal has a stronger resistance to compression deformation and a more stable structure, demonstrating a good permeability recovery rate.

Recommendations for gas extraction in coal seams with different depths are given as follows: (1) for the intact coal seam with shallow buried depth, the matrix rock bridge is often in the elastic deformation stage. The borehole extraction can obtain the opening of fracture to achieve the efficient extraction of methane; (2) with the growing depth of intact coal seam, the geostress increases continuously, and the matrix rock bridge between coal fractures varies from elastic deformation to elastic–plastic deformation. To achieve the efficient extraction, protective layer mining or hydraulic drilling can be used to produce large deformation and matrix damage; (3) in deep coal seam, high geostress causes the plastic deformation or breakage to the majority of matrix rock bridges, and a single stress relief method may not work well. It is necessary to combine protective layer mining and hydraulic punching to achieve the efficient methane extraction; (4) due to the large contact area and strong compression deformation resistance of tectonic coal, elastic deformation may occur only under low stress conditions, and higher stress will cause elastic–plastic deformation of the coal body. However, even under high stress conditions, the fracture is difficult to be completely damaged and destroyed. Therefore, the protective layer mining or other pressure relief methods can achieve a high extraction rate of methane.

## 4. Conclusions

In this study, the permeability characteristics of intact and tectonic coal samples under the process of loading–unloading are tested to investigate the differences in the permeability characteristics, fracture loss degree and the fracture structure. The challenges to the gas extraction in deep coal seams are also discussed. Based on this work, the main findings are summarized as follows:

(1) The permeability recovery of intact coal after high hydrostatic loading–unloading is relatively low, with the rate of only 6.96%, while the unloading permeability of the tectonic coal is much higher than that of the intact coal. The recovery rates of permeability are 40.48% and 20.92%, respectively, for the tectonic coal B and tectonic A.

(2) The main reason for the differences in the permeability characteristics between the intact coal and tectonic coal depends on their fracture structure. The contact area between the matrixes of intact coal is relatively small, and the high stress causes plastic deformation or breakage of the matrix rock bridges. The contact area of tectonic coal matrix is much larger than that of the intact coal, which has a stronger resistance to compression deformation.

(3) With the increase in the depth of a coal seam, the geostress rises continuously. The rock bridges between intact coal fractures gradually transforms from elastic deformation to plastic deformation. The difficulty in the permeability recovery increases continuously, which brings greater challenges to the gas extraction. A variety of stress relief measures should be conducted to realize the effective methane extraction in deep coal seams.

## Conflicts of interest

There are no conflicts to declare.

## Supplementary Material
